# Real-time multispectral fluorescence lifetime imaging using Single Photon Avalanche Diode arrays

**DOI:** 10.1038/s41598-020-65218-3

**Published:** 2020-05-15

**Authors:** João L. Lagarto, Federica Villa, Simone Tisa, Franco Zappa, Vladislav Shcheslavskiy, Francesco S. Pavone, Riccardo Cicchi

**Affiliations:** 1 National Institute of Optics National Research Council (INO-CNR), Largo Enrico Fermi 6, 50125 Florence, Italy; 2 European Laboratory for Non-linear Spectroscopy (LENS), Via Nello Carrara 1, 50019 Sesto Fiorentino, Italy; 3 Dipartimento di Elettronica, Informazione e Bioingegneria (DEIB), Politecnico di Milano, 20133 Milan, Italy; 4 Micro Photon Device SRL, Via Waltraud Gebert Deeg 3g, I-39100 Bolzano, Italy; 5 Becker & Hickl GmbH, Nunsdorfer Ring 7-9, 12277 Berlin, Germany; 6 Department of Physics, University of Florence, Via G. Sansone 1, 50019 Sesto Fiorentino, Italy; 7 Privolzhskiy Medical Research University, 603005 Nizhny Novgorod, Russia

**Keywords:** Biophysics, Engineering, Optics and photonics

## Abstract

Autofluorescence spectroscopy has emerged in recent years as a powerful tool to report label-free contrast between normal and diseased tissues, both *in vivo* and *ex vivo*. We report the development of an instrument employing Single Photon Avalanche Diode (SPAD) arrays to realize real-time multispectral autofluorescence lifetime imaging at a macroscopic scale using handheld single-point fibre optic probes, under bright background conditions. At the detection end, the fluorescence signal is passed through a transmission grating and both spectral and temporal information are encoded in the SPAD array. This configuration allows interrogation in the spectral range of interest in real time. Spatial information is provided by an external camera together with a guiding beam that provides a visual reference that is tracked in real-time. Through fast image processing and data analysis, fluorescence lifetime maps are augmented on white light images to provide feedback of the measurements in real-time. We validate and demonstrate the practicality of this technique in the reference fluorophores and in articular cartilage samples mimicking the degradation that occurs in osteoarthritis. Our results demonstrate that SPADs together with fibre probes can offer means to report autofluorescence spectral and lifetime contrast in real-time and thus are suitable candidates for *in situ* tissue diagnostics.

## Introduction

Optical spectroscopy techniques such as fluorescence, Raman or white light diffuse reflectance have been widely investigated and demonstrated potential for tissue interrogation and surgical guidance in many clinical applications^1–10^. In particular, autofluorescence measurements exploit the fluorescence signatures of endogenous molecules to report the biochemical and structural fingerprints of tissues and thus aim to provide label-free contrast between normal and diseased regions, either by means of single-point spectroscopy^11–13^ or imaging^14–16^.

To date, most clinical studies of tissue autofluorescence are realized in the steady-state regime, by measuring differences in the spectrally-resolved fluorescence signals of normal and diseased tissues. Changes in the autofluorescence spectra of tissues owing to pathological transformations have been reported in osteoarthritis^17^, liver fibrosis^18,19^, cardiovascular applications^20,21^ and many forms of cancer, including in breast^22^, cervix^23^, brain^24^ and skin^25^. While these studies present encouraging results, the specificity of spectrally-resolved autofluorescence measurements is generally limited by the broad overlapping spectra of many tissue fluorophores, such as elastin, collagens, NAD(P)H and flavins^26^. One common method to overcome this limitation is to resolve the autofluorescence measurement both spectrally and temporally, thus increasing the dimension and specificity of the autofluorescence output. Specifically, time-resolved autofluorescence measurements can provide differentiation between endogenous fluorophores with overlapping emission spectra but different fluorescence lifetimes.

For clinical applications, spectral resolution in fluorescence lifetime measurements is typically achieved using a set of dichroic mirrors and optical filters that separate the fluorescence signal in large discrete spectral bands. The resulting spectrally-resolved fluorescence can then be directed to multiple photodetectors^27,28^ or through optical fibres of different lengths that temporally separate the signal from different wavelength bands for detection with a single photomultiplier tube (PMT)^29–32^. Alternative configurations include using monochromators^33–35^, multi-anode PMTs^36–38^ or a filter wheel^39^. Each detection configuration has its own merits and limitations and, thus, their implementation must consider the exact clinical application. For example, while monochromators can provide excellent spectral resolution, they are not suitable for a real-time implementation requiring multispectral lifetime acquisitions, given the long integration times typically required per spectral band to achieve reasonable signal-to-noise ratio (SNR). In this context, large spectral bands are preferred at the expense of spectral resolution. In configurations with multiple discrete detection channels, each with relatively large bandwidth, the spectral range of each channel is defined by the optical transmission of dichroic mirrors and emission filters. This configuration determines that the spectral range and resolution of each channel are fixed and can only be modified by changing filters and/or dichroic mirrors in the detection system. Accordingly, these instruments are typically configured to include large detection channels that overlap with spectral regions where common endogenous fluorophores emit most strongly. This strategy necessarily limits the specificity and versatility of the instrument: if additional spectral range or resolution is required, the entire detection system would have to be modified.

In this context, Single Photon Avalanche Diode (SPAD) arrays have emerged as attractive alternatives to common multispectral lifetime strategies, given that spectral and temporal resolution to the fluorescence measurement can be provided and adjusted at the sensor level using a fixed optical detection setup^40–42^. While the base technology is still at relatively early stage of development, recent studies have demonstrated the potential of SPADs in biomedical research, including super-resolution microscopy^43^, measurements of protein-protein interactions^44^ and molecular-based diagnosis^45^. In this study, we extend the application of SPAD arrays to single-point measurements, to realize multispectral fluorescence lifetime imaging using fibre optic probes. Specifically, this study expands our previous work^46^, where we demonstrated the feasibility of generating fluorescence lifetime maps from single-point measurements at a macroscopic scale and under bright illumination of the specimen using Time-Correlated Single Photon Counting (TCPSC) instrumentation. Here, we explore the versatility of SPAD arrays to realize multispectral fluorescence lifetime acquisitions in real-time using a single-point approach. To enhance potential for clinical deployment, fluorescence intensity and lifetime data are overlaid on white light images acquired with an external RGB CMOS camera, thereby providing spatial and fluorescence feedback in real-time. We present a comprehensive description and characterization of this method, including its validation in articular cartilage samples presenting local degradation following enzymatic digestion of collagen. We believe this technique can provide rapid multidimensional information that could aid tissue diagnosis and thus could have significant impact in many clinical applications.

## Methods

### Experimental setup

#### Optical setup

**Figure 1:**
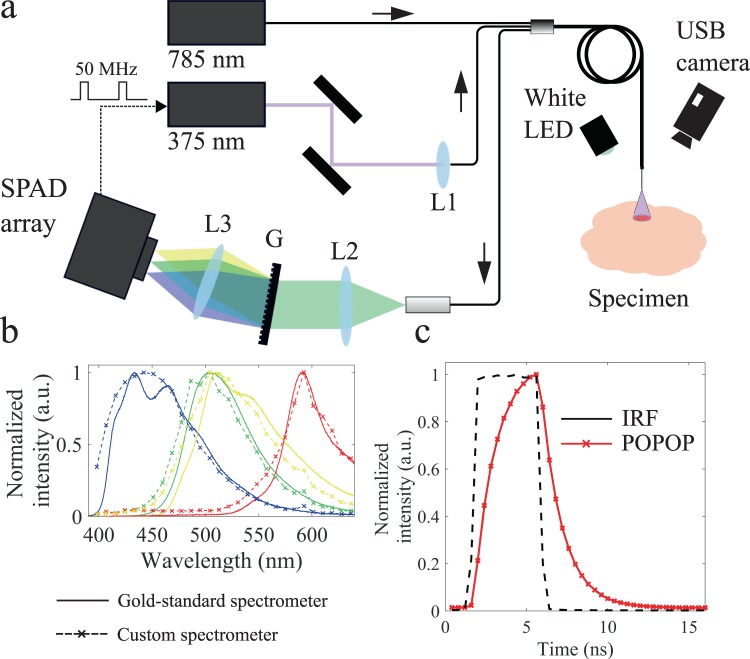
(**a**) Optical layout of the multispectral fluorescence lifetime imaging system. Fluorescence light is dispersed and spectrally-resolved across the long axis of the SPAD array using a transmission grating comprising 600 grooves/mm. L1: Aspheric lens, f = 11.0 mm (A220TM, Thorlabs); L2 and L3: Bi-Convex Lens, f = 60.0 mm (LB1723-B, Thorlabs). (**b**) Fluorescence emission spectra of reference samples measured with a Horiba microHR monochromator and a Syncerity CCD camera (solid lines) and our instrument (crossed dashed lines). (**c**) Instrument response function and representative fluorescence intensity decay of a reference fluorophore (50 μM POPOP in ethanol, τ = 1.3 ns).

Experiments carried out in this study were realized using a custom-built fibre-based multispectral fluorescence lifetime imaging setup (see Fig. 1a) and partially described elsewhere^42^. Excitation light at 375 nm was provided by a laser diode (BDL-SMN-375, Becker & Hickl GmbH, Berlin, Germany) that produced short optical pulses (<100 ps) at 50 MHz repetition rate. Excitation light was delivered to the sample via a 200 μm core diameter optical fibre embedded in a quadrifurcated fibre bundle (NA = 0.22, length = 4 m, EMVision LLC, Loxahatchee, FL, USA). The average power at the sample plane was kept below 25 μW at all times during measurements, which is below the maximum permissible exposure (MPE) for skin and eye. At a probe-to-target distance of 3 mm, this corresponds to a power density of less than 5 mW/cm^2^, which is similar or less than that used for excitation in previous *in vivo* or *ex vivo* investigations, using similar excitation strategies^36–38,47^. In none of these studies alterations in the biological characteristics of the sample as a result of laser excitation were reported. The fluorescence signal emanating from the sample was collected by seven 300 μm 0.22 NA optical fibres and delivered to the detection system. In the detection system, fluorescence light was dispersed by a transmission grating (GT50-06V, Thorlabs, Newton, NJ, USA) and the resulting spectrally-resolved fluorescence signal imaged with 1:1 magnification across the long axis of a SPAD array (SPC3, MPD, Bolzano, Italy) consisting of 2048 pixels arranged in 32 rows by 64 columns. A 400 nm long-pass filter (FEL0400, Thorlabs) was added to the emission path to prevent excitation light to reach the detector. We chose this optical configuration given its simplicity and broad wavelength range (400–650 nm) for detection.

#### Augmented fluorescence lifetime and spectral imaging in real time

To generate autofluorescence lifetime and intensity maps out of single-point measurements, we used the aiming beam approach that was previously described by others^30^ and expanded by our group in the context of TCSPC measurements^46^. In brief, fluorescence measurements are realized by moving the fibre probe freely across the specimen. A second light source - a continuous-wave (CW) 785 nm laser diode (FC-785-350-MM2-PC-0-RM, RGBLase, Fremont, CA, USA) - is used simultaneously with the 375 nm excitation light, within the same fibre bundle but through an adjacent 200 μm core. The localization of the fluorescence excitation and detection is done by tracking the 785 nm beam in real time using a colour camera (DFK 33UP1300, The Imaging Source, Bremen, Germany). To prevent contamination of the fluorescence signal with 785 nm light, a 700 nm short-pass filter (ET700SP, Chroma Technologies, Bellows Falls, VT, USA) was also added to the detection path.

A significant limitation of photon counting approaches within the visible region of the spectrum refers to their inability to discern fluorescence from background photons (e.g. ambient room light). To overcome this limitation, instead of ambient light, a modulated LED (LED, MNWHL4, Thorlabs) was used to illuminate the field-of-view (FOV) of a sample. The modulation was performed at 50 Hz and 10% duty cycle, resulting in 2 ms illumination time, with an illuminance of approximately 200 LUX. At this frequency, the stroboscopic effect produced by the on-off switching of the LED is not perceived by the human eye and the FOV will appear to be continuously illuminated to the operator. In turn, fluorescence lifetime and spectral measurements are realized at 50 Hz, but within the non-illuminated cycle of the LED, i.e. in the remaining 18 ms. This method guarantees that fluorescence measurements are free from background light originated by the pulsing LED and, thus, from the operator’s perspective, are realized under bright illumination of the FOV. Finally, triggering the colour camera simultaneously with the LED will produce well-illuminated white light images at 50 Hz (with ~2 ms exposure time), which can be used for determining the interrogated point in real-time, as described above. Given that camera and fluorescence acquisitions are synchronized, each fluorescence measurement is associated with a position of the fibre probe. In consequence, fluorescence maps can be generated to provide visual feedback of the measurements in real-time. One particular highlight of this method is that spectral bands can be selected and tuned on-the-fly during processing, so that online feedback is provided for a specific region of the spectrum, and the spectral band can be changed if necessary.

#### SPAD array and photon time-gating strategy

The sensor used in our experiments is an array of 32 × 64 SPADs (4.8 mm × 9.6 mm) developed using 0.35 µm complementary metal-oxide semiconductor (CMOS) technology. The pixel size is 150 µm × 150 µm, and the SPAD diameter is 30 µm, which results in a fill-factor of 3.14%. The detector features a peak Photon Detection Efficiency (PDE) of 50% at 450 nm and a median Dark Counting Rate (DCR) of 150 cps per pixel. The dead time in each pixel is 50 ns. A complete characterization of the SPAD detector is provided in references^48,49^. Histogram of photon arrival times are generated independently in each pixel by means of time-gating^50,51^, at a fixed repetition rate of 50 MHz, which was used for synchronization with the excitation laser. Measurements of the instrument response function (IRF) and fluorescence intensity decays of a fluorescence standard using different gating configurations are illustrated in Supplementary Fig. S1.

Considering the specificities of our implementation, as described above, the specific photon time-gating strategy must balance a number of parameters, namely: (1) photon integration time, since a single fluorescence acquisition must necessarily occur within the non-illuminated period of the white LED (i.e. maximum of 18 ms at 50 Hz with 10% LED duty cycle); (2) photon collection efficiency; (3) temporal resolution. Since the integration time of our application is limited, in order to improve photon collection efficiency while maintaining reasonable sampling of the fluorescence intensity decays, we chose a detection strategy employing long overlapping gates (4 ns width). The detection gate scanned the fluorescence decay in 400 ps steps, for a total acquisition window of 16 ns and 40 sampling points. All measurements presented in this study were realized with these gate settings. Representative fluorescence lifetime and IRF curves are shown in Fig. 1c. For the gate settings used in this study, the measured IRF full-width at half maximum (FWHM) was 4.30 ± 0.04 ns.

### Data analysis

#### Fluorescence lifetime data

To provide feedback of the measurements in real-time, fluorescence lifetime data are analysed immediately upon each single acquisition using the modified phasor approach for time-gated fluorescence lifetime measurements, which is described in detail elsewhere^52^. In brief, each measured fluorescence intensity decay is Fourier Transformed to obtain the corresponding phasor coordinates *g* and *s*. In practice, the measured fluorescence signal is a convolution of the sample’s fluorescence response and IRF. The contribution of the IRF can be accounted for by calibrating the measurement using a reference sample, which corresponds to an additional step of rescaling and rotation in the phasor transformation. In our measurements, we used the back-reflected signal measured from excitation light as calibration (τ = 0 ns). Following phasor transformation and calibration, the characteristic phasor of single-exponential emitters falls on the universal circle. A mixture of two fluorophores with distinct single-exponential characteristics lies inside the universal circle, as a linear combination of each individual component. Hence, any combination of the two fluorophores falls along a line connecting their characteristic single-exponential phasors. From the coordinates *g* and *s*, phase (*τ*_*phase*_) and modulation (*τ*_*mod*_) lifetimes can be calculated. In the measurements below, we report *τ*_*phase*_, which is given by *τ*_*phase*_ = tan *φ /ω*, where *φ* is the angle between the phasor vector and the x-axis (i.e. tan *φ* = *s/g*), and *ω* is the angular frequency (*ω = 2πf*), where *f* is the laser repetition rate, i.e. 50 MHz).

Given the high repetition rate of the excitation laser (50 MHz, 20 ns periods) and comparatively short acquisition window (16 ns), the recorded fluorescence decay curves may include contributions from previous excitation periods, particularly for slow decays. Our analysis model does not account for such incomplete recordings, which may lead to slight inaccuracies in the estimation of long fluorescence lifetimes. For two references fluorophores (POPOP in ethanol and flavin adenine dinucleotide (FAD) in purified water) we measured fluorescence lifetimes of 1.36 ± 0.04 ns and 4.02 ± 0.08 ns, respectively. These are comparable to fluorescence lifetimes of 1.32 ± 0.03 ns and 3.79 ± 0.06 ns measured in a TCSPC setup^46^.

#### Fluorescence intensity data

Fluorescence spectra were normalized by dividing the fluorescence intensity measured at each wavelength by the peak intensity. For measurements where data were binned in large spectral bands, the autofluorescence intensity in each band was calculated as a fraction of the total autofluorescence signal, i.e. the sum of all detection channels. Spectral calibration was realized by measuring the back-reflected signal from a 445 nm laser diode (Sacher Lasertechnik GmbH, Marburg, Germany) and LEDs with center wavelength at 470 nm, 530 nm and 630 nm. Spectral measurements of calibration LEDs and laser were initially realized using a gold-standard microHR monochromator (Horiba, Kyoto, Japan) with a Syncerity CCD detector (Horiba) and used to calibrate our custom spectrometer. Spectral measurements of fluorescent slides (Chroma Technologies Corporation, Bellows Falls, VT, USA) were compared and showed a discrepancy of approximately 5 nm between systems, which is equivalent to the spectral resolution of our custom implementation (see Fig. 1b).

### Porcine articular cartilage digestion

Articular cartilage specimens (approximate dimensions 3 cm × 3 cm × 3 cm) were obtained from metacarpophalangeal joints of freshly slaughtered pigs and kept in PBS with 0.05% sodium azide for 24 hours to prevent bacterial growth. Samples were thoroughly washed in Phosphate Buffer Saline (PBS) and kept at −20 °C for 24 hours before measurements. Articular cartilage digestion was induced using filter paper soaked in 250 μg/mL bacterial collagenase on the articular surface, as illustrated in Supplementary Fig. S2. Treatment was applied for 5 hours at 37 °C. Samples were thoroughly washed in PBS after treatment to remove any residues of digestive enzyme. Fluorescence measurements were realized before and after treatment over the entire cartilage surface to demonstrate spatial variation of the fluorescence signal.

## Results

To demonstrate our implementation in clinically relevant biological tissue, we measured the autofluorescence lifetime and spectral signatures of porcine articular cartilage. Measurements were realized before and after treatment of the articular surface with bacterial collagenase, to induce endogenous contrast derived from the localized digestion of collagen and consequent alteration of its fluorescence characteristics, in an effort to mimic natural degradation occurring in osteoarthritis^47,53^. The photon integration time for each autofluorescence acquisition was set to ~11 ms. For this temporal window, we measured a negligible contribution from background light originating from the LED (see Supplementary Fig. S3), and fluorescence lifetime and image acquisition rates of 24.94 ± 0.03 Hz and 33.33 ± 1.33 Hz, respectively. These acquisition rates are slower that the expected 50 Hz, and this difference results from delays in fluorescence lifetime and image acquisition and analysis that slows down concurrent processes. For 13 ms integration time, we also measured a negligible number of photons associated to background LED light, but the decrease in acquisition rates was more pronounced (see Fig. S4). For integration times longer than 15 ms, there is an increase in the contribution of LED light to the autofluorescence measurement (~20%, see Fig. S3), which also results from delays in data acquisition and processing that cause the fluorescence acquisition to overlap with the illumination cycle of the LED.

**Figure 2:**
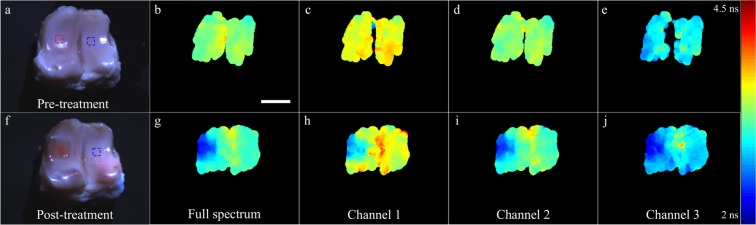
Autofluorescence lifetime maps of cartilage pre- (top row) and post-treatment (bottom row) with bacterial collagenase. (**a,f**) White light images of the cartilage specimen. (**b, g**) Fluorescence lifetime maps generated by binning the entire array into a single channel. Panels (**c–e**) and (**h–j**) show fluorescence lifetime maps for different wavelength bands: channel 1 (400–450 nm); channel 2 (450–515 nm); channel 3 (515–580 nm). Wavelengths bands were generated in post-processing by spectrally binning 15 columns of pixels. Scale bar = 10 mm.

**Figure 3:**
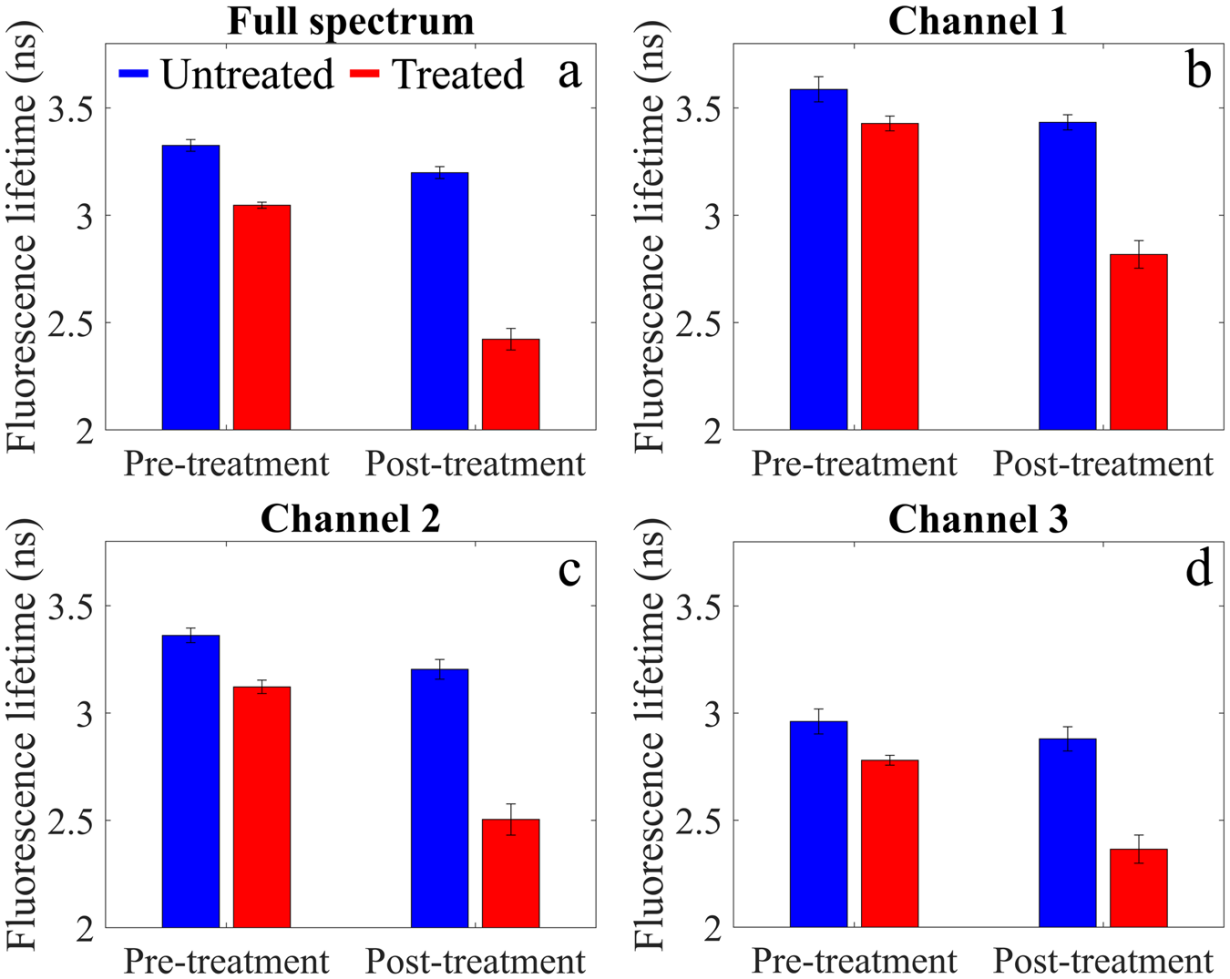
Average fluorescence lifetimes for each spectral channel in regions of interest (ROI), as illustrated in Fig. 2(a,f).

**Figure 4:**
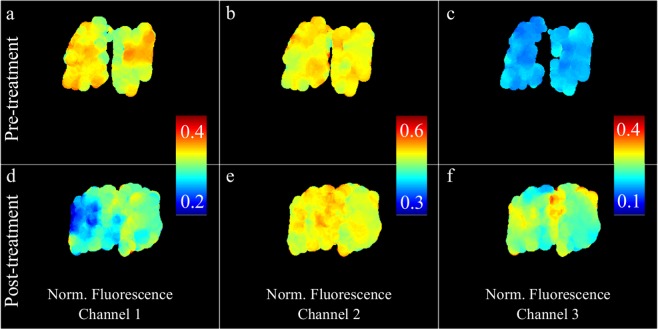
Normalized fluorescence intensity maps of cartilage pre- (top row) and post-treatment (bottom row) with bacterial collagenase. (**a,d**) Channel 1 (400–450 nm); (**b,e**) channel 2 (450–515 nm); (**c,f**) channel 3 (515–580 nm).

Autofluorescence lifetime of porcine articular cartilage pre- and post-treatment with bacterial collagenase are presented in Figs. 2 and 3 for the entire spectral range and for distinct spectral bands: channel 1, 400–450 nm (Fig. 2(c,h)); channel 2, 450–515 nm (Fig. 2(d,i)); channel 3, 515–580 nm (Fig. 2(e,j)). In the spectral region 580–650 nm, we measured a weak fluorescence signal and thus data for this band are not shown. The normalized autofluorescence intensity maps are shown in Fig. 4. Demonstrations of the measurements are also presented in Supplementary Videos S1–S3. We note that, during acquisition, online feedback was provided for the entire spectral range (see Supplementary Video S1).

Before treatment (see Fig. 2(a–e)), we observed a small variation of the autofluorescence lifetime across the articular surface (see Fig. 3), possibly arising from regions with different load-bearing characteristics. In the region where treatment was applied (see Fig. 2a, red square, n = 1088 pixels), we measured pre-treatment average autofluorescence lifetimes of 3.44 ± 0.03 ns, 3.20 ± 0.04 ns and 2.89 ± 0.05 ns, for the wavelengths bands of channels 1, 2 and 3, respectively, which indicate a slight dependency of the autofluorescence lifetime with wavelength. If we merge the data in a single spectral band (Figs. 2b and 3a), we obtain an average lifetime of 3.20 ± 0.05 ns.

**Figure 5:**
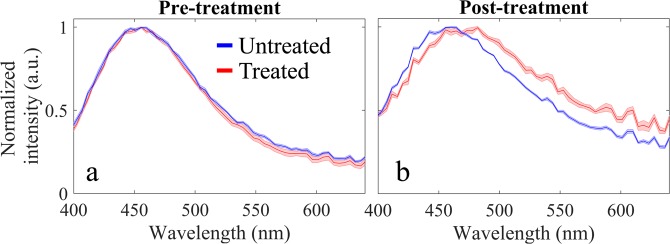
Average fluorescence emission spectra (left) pre and (right) post enzymatic treatment in regions of interest (ROI), as illustrated in Fig. 2(a,f). Solid lines indicate average spectra and shaded regions the corresponding standard deviation.

Treatment with bacterial collagenase resulted in visible digestion of the articular cartilage surface (see Fig. 2f, red square), which, in turn, resulted in a consistent decrease of autofluorescence lifetime in this region, as compared to non-digested cartilage. While a decrease in autofluorescence lifetime is visible in all spectral bands, this is most evident in channel 2 (see Fig. 2h), which is coincident with the fluorescence emission peak of collagen (see Fig. 5). We also measured a slight decrease in the autofluorescence lifetime of the non-digested region, although this is not as evident as in the region of collagen digestion. In the region of digestion (n = 1740 pixels), we measured autofluorescence lifetimes of 2.82 ± 0.06 ns, 2.51 ± 0.07 ns and 2.36 ± 0.07 for channels 1, 2 and 3, respectively. For the entire spectral range, we measured an average lifetime of 2.42 ± 0.05 ns.

With respect to autofluorescence intensity (Fig. 4), our data suggest a red shift in the autofluorescence spectrum of the digested region relative to non-digested cartilage. This is most visible in Fig. 4d, as a decrease in the fraction of autofluorescence emanating from channel 1, but also in the average autofluorescence spectra, from which we measured a shift in the peak emission wavelength of approximately 10 nm (see Fig. 5b). We note that, in general, we measured a lower absolute autofluorescence signal after treatment, which generated noisier autofluorescence maps and spectra.

## Discussion

In a previous study, we introduced a technique employing TCSPC and hybrid photon counting detectors to realize single-channel fibre-based fluorescence lifetime imaging from single-point measurements in real-time and under bright illumination of the FOV^46^. Here, we aimed to expand this technique by employing SPAD array detectors to provide multispectral resolution to the lifetime measurement and thus increase the specificity of the fluorescence output. As in our previous study with a TCSPC-based setup, interleaving the fluorescence acquisition with external illumination from a pulsed white LED at 50 Hz permitted single-point measurements to be realized at a sufficiently high rate (between 25 and 50 Hz, see Supplementary Fig. S4) to generate fluorescence lifetime and intensity maps in real-time. We note that while measurements were software triggered at 50 Hz, timing delays in data processing and storage following acquisition slowed down the rate at which fluorescence data were acquired. To minimize interference of timing delays in acquisition rate, the integration time for fluorescence acquisitions was adjusted to ~11 ms. Considering the poor photon efficiency of our system, as further discussed below, we aimed for the longest possible integration time within the LED non-illuminated cycle. However, increasing the integration time beyond 11 ms consistently caused the fluorescence acquisition to overlap with the illumination cycle of the LED, thus leading to detection of undesirable LED-background (see Supplementary Fig. S3). For integration times longer than 15 ms, the contribution of LED background to the measured signal was approximately 20%. While such contribution is not sufficient to mask the autofluorescence signal from the sample, the LED illuminance in our measurements was approximately 200 LUX, which is about 10–20% of the illuminance provided by an endoscopic system. Thus, under standard illumination from an endoscope, the background contribution would not be negligible.

The white LED guarantees that the sample is visible to the human eye and sufficiently illuminated during measurements. From the operator’s perspective, the LED provides continuous illumination of the FOV, since 50 Hz switching frequency cannot be perceived by the human eye. This is a key element in our implementation towards clinical translation. So far, clinical translation of optical techniques dedicated to surgical guidance and operating in the visible range of the spectrum has been limited by the intrinsic inability to discern fluorescence photons from ambient background light. Indeed, a number of studies can be found in literature employing analogue detection methods that separate the low frequency background from the high frequency fluorescence decays^34,54,55^. However, such separation is not trivial in photon counting techniques; thus the applicability of such measurements in clinical studies is limited. Our technique, while still requiring room lights to be turned off, allows fluorescence measurements in the visible spectrum to be realized together with bright LED illumination, in a relatively straightforward implementation. This makes our technique compatible with many clinical procedures where LED illumination is becoming standard, such as endoscopy, arthroscopy or robot-assisted surgery, requiring only minimal modifications to the existing clinical setups.

A key point of our implementation refers to the dynamic spectral range for fluorescence lifetime determination that can be tuned according to any specific target. The ability to dynamically modify the range of wavelengths over which fluorescence lifetimes are measured constitutes a great advantage over traditional multichannel instruments with fixed detection spectral range, which are defined by the combined optical transmission of dichroic mirrors and emission filters e.g^27,30,56^. In our implementation, fluorescence is passed through a transmission grating and spectrally-resolved across 64 columns of SPAD detectors, between ~400 nm and 650 nm. If the SNR in one spectral band at maximum resolution is not sufficient to provide robust fluorescence lifetime measurements in real-time, the spectral bandwidth can be increased to improve the SNR by merging adjacent columns of pixels, at the expense of spectral resolution (see Supplementary Video S3). Additionally, data can be spectrally combined to target the emission bands of fluorophores of interest, for any particular clinical application (see Supplementary Video S2). For example, in applications requiring assessment of tissue metabolic properties, the spectral range can be tuned to the bands of key endogenous metabolites NAD(P)H and FAD, i.e. 430–470 nm and 500–550 nm, respectively. In other applications requiring e.g. monitoring collagen autofluorescence, the spectral range can be modified in order to maximize collagen autofluorescence detection, i.e. from 400–460 nm. If additional fluorescence specificity is required, e.g. separate out free- and protein-bound NAD(P)H or to target porphyrin autofluorescence, different spectral bands can be dynamically generated, from which spectrally-resolved fluorescence lifetime data can be retrieved. The benefits of having dynamic spectral range and resolution are also illustrated in Fig. S5c, where we measured the fluorescence lifetime characteristics of two reference fluorophores and mixture solutions. Specifically, our results demonstrate that increasing the number of pixels in each spectral channel, i.e. lower spectral resolution, decreases the specificity of the fluorescence lifetime output, as expected. In summary, in our implementation, spectral bands for lifetime determination can be dynamically selected before or during the acquisition, or in post-processing, and must balance the required specificity for the measurements and the amount of signal at key spectral bands: if one spectral band has low SNR, the spectral range can be increased at the expense of spectral resolution.

The feasibility and potential of our approach to report autofluorescence contrast was demonstrated in cartilage specimens. Specifically, the articular surface of porcine metacarpophalangeal joints was digested with bacterial collagenase, in an effort to mimic extracellular matrix degradation that is characteristic of osteoarthritis (OA)^57,58^. We chose this particular application given the general interest in identifying comprehensive and robust solutions to diagnose and monitor the early onset of OA, which are, to date, inexistent. While the onset of OA is believed to be driven by mechanical factors, some studies suggested that early stages of disease progression are dominated by biochemical alterations in tissue^59^, which are not detectable using conventional diagnostic modalities, such as x-ray and magnetic resonance imaging, but make multispectral fluorescence lifetime imaging a suitable candidate technology to detect early development of OA. Overall, our autofluorescence spectral and lifetime data (see Figs. 2–5) reveal some level of heterogeneity in the fluorescence characteristics of normal articular cartilage. This is most evident in the normalized autofluorescence intensity measured in channel 1 (see Fig. 4a), where lateral regions of the joint appear to have a stronger emission compared to medial regions. Variations in the autofluorescence signature of normal articular cartilage may be associated with variations in load-bearing characteristics across the joint. Autofluorescence lifetime maps generated for different spectral bands (see Fig. 2) reveal a well-demarcated region that is coincident with the area of collagen digestion, yielding shorter lifetime relative to non-digested areas. These results are in close agreement with previous studies of cartilage digestion^46,47,53^. The decrease in autofluorescence lifetime is more prominent in channels 1 and 2, i.e. from 400 to 515 nm, and coincident with the measured fluorescence emission peak (see Fig. 5). Interestingly, our results suggest that autofluorescence lifetime of cartilage decreases slightly with wavelength (~500 ps from 400 to 650 nm), which is consistent with previous observations^60^. While collagen is the dominant source of autofluorescence in cartilage, it is possible that other fluorophores contribute slightly to the autofluorescence signal at longer wavelengths where the contribution of collagen is smaller, namely glycosaminoglycans^61^, and produce an overall decrease in lifetime with wavelength. This could also explain the smaller decrease in lifetime in channel 3 observed upon digestion of the articular surface (see Figs. 2 and 3d). Changes in the autofluorescence properties of articular cartilage upon digestion with bacterial collagenase were also evident in the fluorescence emission spectra, where we measured a consistent red shift in the fluorescence emission of digested cartilage relative to non-digested and pre-treated cartilage. This is visible in the normalized autofluorescence intensity maps (see Fig. 4) and in the autofluorescence emission spectra (see Fig. 5) averaged over the highlighted ROIs (see Fig. 2(a,f) for identification of ROIs). Specifically, spectral measurements show a ~10 nm shift towards longer wavelengths in the fluorescence emission of digested cartilage relative to non-digested cartilage (see Fig. 5), which is in agreement with previous studies^17,47^ but directly contradicts another^53^.

One current limitation of this system refers to its low photon efficiency. This is of paramount importance for real-time implementations, since the photon integration time is limited by the overall timing requirements of the application. To some extent, for autofluorescence measurements presented in this study the issue of low photon efficiency was mitigated by binning adjacent columns of pixels to increase SNR, at the expense of spectral resolution. Furthermore, our measurements were realized in collagenous tissue, which typically exhibits strong autofluorescence signal due to the high quantum yield of collagen. In tissues presenting weak autofluorescence signal, as is common, the low detection efficiency could potentially limit the viability of the system for real-time implementations and, therefore, needs addressing in future investigations. A number of factors contribute to the overall poor photon efficiency. 1) The time-gated strategy for fluorescence lifetime measurements is intrinsically inefficient, given that the photon collection window only covers a small percentage of the total acquisition period (20% in our measurements), and all photons arriving at the detector outside the collection window are discarded. Alternative strategies could employ multiple parallel windows to realize fluorescence acquisition with 100% duty cycle^62,63^. 2) The fill factor of each SPAD detector is low (<4%^48^) and can potentially be optimized using microlenses. 3) There are significant losses in the detection arm, given the relatively low efficiency of the transmission grating (~55% at 550 nm) and lenses. To improve light throughput, the spectrometer could be designed using spherical mirrors and a reflective grating, which is a significantly more efficient design compared to ours^64^.

Our system is most sensitive to fluorescence signals emitted from the most superficial layers of tissues, due to the strong tissue scattering and absorption at the excitation and emission wavelengths used in this study, that limit the region of interrogation to the first 100–200 μm depth. This can be a disadvantage in cases where disease processes vary with depth and extend to deeper layers, as is the case of some tumours. To some extent, depth information can be provided by resolving the fluorescence emitted from different layers across the rows of the SPAD array, as previously described^42^. In addition, multispectral fluorescence lifetime measurements could be employed together with a technique that can harness information from deep layers of tissues, such as diffuse reflectance^37^, Raman spectroscopy^65^ or Optical Coherence Tomography^66^.

In conclusion, we presented an exploitation of SPAD arrays to realize multispectral fluorescence lifetime imaging in real-time by means of fibre optic probes and under bright visible illumination of the FOV, thus expanding on our previous work using single-channel TCSPC-based instrumentation. In particular, we demonstrated the versatility of SPAD arrays to provide dynamic spectral range and resolution to fluorescence lifetime measurements, which constitutes an advantage over traditional multichannel instrumentation. This can be of interest in tissue measurements with multiple contributing endogenous fluorophores, thus requiring additional specificity to resolve the various fluorescence emitters. Given the simplicity of our implementation to produce multidimensional fluorescence images out of single-point measurements, we believe this method has potential to provide real-time diagnostic information and guidance in many clinical imaging modalities, such as arthroscopy, colonoscopy, endoscopy or robot-assisted surgery.

## Supplementary information

Supplementary Information.

Supplementary Information2.

Supplementary Information3.

Supplementary Information4.

## References

[1] Liu, C. H. *et al*. Raman, fluorescence, and time-resolved light scattering as optical diagnostic techniques to separate diseased and normal biomedical media. *J. Photochem. Photobiol. B.* **16**, 187–209 (1992). 10.1016/1011-1344(92)80008-j 1474426

[2] Zonios, G. *et al*. Diffuse reflectance spectroscopy of human adenomatous colon polyps *in vivo. Appl. Opt.* **38**, 6628–37 (1999). 10.1364/ao.38.006628 18324198

[3] Ramanujam N (2000). Fluorescence spectroscopy of neoplastic and non-neoplastic tissues. Neoplasia.

[4] Hanlon, E. B. *et al*. Prospects for *in vivo* Raman spectroscopy. *Phys. Med. Biol.* **45**, R1–59 (2000). 10.1088/0031-9155/45/2/201 10701500

[5] Huang, Z., Lui, H., McLean, D. I., Korbelik, M. & Zeng, H. Raman spectroscopy in combination with background near-infrared autofluorescence enhances the *in vivo* assessment of malignant tissues. *Photochem. Photobiol.* **81**, 1219–26 (2005). 10.1562/2005-02-24-RA-449 15869327

[6] Brown CP (2009). Diffuse reflectance near infrared spectroscopy can distinguish normal from enzymatically digested cartilage. Phys. Med. Biol..

[7] Bergholt MS (2011). Combining near-infrared-excited autofluorescence and Raman spectroscopy improves in vivo diagnosis of gastric cancer. Biosens. Bioelectron.

[8] Johansson A, Sundqvist T, Kuiper J-H, Öberg PÅ (2011). A spectroscopic approach to imaging and quantification of cartilage lesions in human knee joints. Phys. Med. Biol..

[9] Spliethoff JW (2013). Improved identification of peripheral lung tumors by using diffuse reflectance and fluorescence spectroscopy. Lung Cancer.

[10] Jafari MD (2013). The use of indocyanine green fluorescence to assess anastomotic perfusion during robotic assisted laparoscopic rectal surgery. Surg. Endosc. Other Interv. Tech.

[11] De Veld DCG, Witjes MJH, Sterenborg HJCM, Roodenburg JLN (2005). The status of in vivo autofluorescence spectroscopy and imaging for oral oncology. Oral Oncol..

[12] Wu Y, Qu JY (2006). Autofluorescence spectroscopy of epithelial tissues. J. Biomed. Opt..

[13] Lukina M (2017). Interrogation of metabolic and oxygen states of tumors with fiber-based luminescence lifetime spectroscopy. Opt. Lett..

[14] Andersson-Engels S, Klinteberg C, Svanberg K, Svanberg S (1997). *In vivo* fluorescence imaging for tissue diagnostics. Phys. Med. Biol..

[15] Lin, D. *et al*. Autofluorescence and white light imaging-guided endoscopic Raman and diffuse reflectance spectroscopy for *in vivo* nasopharyngeal cancer detection. *J. Biophotonics* 1–9, 10.1002/jbio.201700251 (2018). 10.1002/jbio.201700251 29239125

[16] Shcheslavskiy VI (2018). Fluorescence time-resolved macroimaging. Opt. Lett..

[17] Lewis, W., Padilla-Martinez, J. P., Ortega-Martinez, A. & Franco, W. Changes in endogenous UV fluorescence and biomechanical stiffness of bovine articular cartilage after collagenase digestion are strongly correlated. *J. Biophotonics* **8**, 1–8 (2016). 10.1002/jbio.201600093 27714971

[18] Arroyo-camarena, U. D., Dura, M. A., Ibarra-coronado, E. & Herna, L. F. Fluorescence Spectroscopy as a Tool for the Assessment of Liver Samples with Several Stages of Fibrosis. **36**, 151–161 (2018). 10.1089/pho.2017.4301 29131710

[19] Nazeer, S. S., Saraswathy, A., Shenoy, S. J. & Jayasree, R. S. Fluorescence spectroscopy as an efficient tool for staging the degree of liver fibrosis: an *in vivo* comparison with MRI. *Sci. Rep* **8**, 1–11 (2018). 10.1038/s41598-018-29370-1 PMC6054616 30030510

[20] Ti Y, Chen P, Lin W-C (2010). *In vivo* characterization of myocardial infarction using fluorescence and diffuse reflectance spectroscopy. J. Biomed. Opt..

[21] Lagarto JL (2019). *In vivo* label-free optical monitoring of structural and metabolic remodeling of myocardium following infarction. Biomed. Opt. Express.

[22] Tang GC (1989). Pradhan, a & Alfano, R. R. Spectroscopic differences between human cancer and normal lung and breast tissues. Lasers Surg. Med..

[23] Cardenas-Turanzas M (2007). The clinical effectiveness of optical spectroscopy for the *in vivo* diagnosis of cervical intraepithelial neoplasia: where are we?. Gynecol. Oncol..

[24] Liu Q (2011). Compact point-detection fluorescence spectroscopy system for quantifying intrinsic fluorescence redox ratio in brain cancer diagnostics. J. Biomed. Opt..

[25] Lohmann, W. & Paul, E. *In situ* detection of melanomas by fluorescence measurements. *Naturwissenschaften* **75**, 201–202 (1988). 10.1007/BF00735581 3398925

[26] Wagnieres GA, Star WM, Wilson BC (1998). *In vivo* Fluorescence Spectroscopy and Imaging for Oncological Applications. Photochem. Photobiol.

[27] Lagarto J (2015). Application of time-resolved autofluorescence to label-free *in vivo* optical mapping of changes in tissue matrix and metabolism associated with myocardial infarction and heart failure. Biomed. Opt. Express.

[28] Lagarto JL (2018). Characterization of NAD(P)H and FAD autofluorescence signatures in a Langendorff isolated-perfused rat heart model. Biomed. Opt. Express.

[29] Yankelevich DR (2014). Design and evaluation of a device for fast multispectral time-resolved fluorescence spectroscopy and imaging. Rev. Sci. Instrum.

[30] Gorpas D, Ma D, Bec J, Yankelevich D, Marcu L (2016). Real-Time Visualization of Tissue Surface Biochemical Features Derived from Fluorescence Lifetime Measurements. IEEE Trans. Med. Imaging.

[31] Bec, J. *et al*. *In vivo* label-free structural and biochemical imaging of coronary arteries using an integrated ultrasound and multispectral fluorescence lifetime catheter system. *Sci. Rep* **7**, 8960–9 (2017). 10.1038/s41598-017-08056-0 PMC5566546 28827758

[32] Lagarto, J. L. *et al*. Electrocautery effects on fluorescence lifetime measurements: An *in vivo* study in the oral cavity. *J. Photochem. Photobiol. B Biol* **185**, 90–99 (2018). 10.1016/j.jphotobiol.2018.05.025 PMC6629261 29883910

[33] Ashjian P (2004). Noninvasive *in situ* evaluation of osteogenic differentiation by time-resolved laser-induced fluorescence spectroscopy. Tissue Eng..

[34] Butte PV (2011). Fluorescence lifetime spectroscopy for guided therapy of brain tumors. Neuroimage.

[35] Sun Y (2012). Nondestructive Evaluation of Tissue Engineered Articular Cartilage Using Time-Resolved Fluorescence Spectroscopy and Ultrasound Backscatter Microscopy. Tissue Eng. Part C Methods.

[36] De Beule PAA (2007). A hyperspectral fluorescence lifetime probe for skin cancer diagnosis. Rev. Sci. Instrum.

[37] Thompson, A. J. *et al*. *In vivo* measurements of diffuse reflectance and time-resolved autofluorescence emission spectra of basal cell carcinomas. *J. Biophotonics* **5**, 240–54 (2012). 10.1002/jbio.201100126 22308093

[38] Coda S (2014). Fluorescence lifetime spectroscopy of tissue autofluorescence in normal and diseased colon measured *ex vivo* using a fiber-optic probe. Biomed. Opt. Express.

[39] Poulon F (2017). Optical properties, spectral, and lifetime measurements of central nervous system tumors in humans. Sci. Rep.

[40] Krstajić N, Levitt J, Poland S, Ameer-beg S, Henderson R (2015). 256 × 2 SPAD line sensor for time resolved fluorescence spectroscopy. Opt. Express.

[41] Popleteeva M (2015). Fast and simple spectral FLIM for biochemical and medical imaging. Opt. Express.

[42] Lagarto JL (2019). Multispectral Depth-Resolved Fluorescence Lifetime Spectroscopy Using SPAD Array Detectors and Fiber Probes. Sensors.

[43] Castello M (2019). A robust and versatile platform for image scanning microscopy enabling super-resolution FLIM. Nat. Methods.

[44] Poland SP (2014). Time-resolved multifocal multiphoton microscope for high speed FRET imaging *in vivo*. Opt. Lett..

[45] Canals J, Franch N, Alonso O, Vilà A, Diéguez A (2019). A Point-of-Care Device for Molecular Diagnosis Based on CMOS SPAD Detectors with Integrated Microfluidics. Sensors.

[46] Lagarto, J. L., Shcheslavskiy, V., Pavone, F. S. & Cicchi, R. Real-time fiber-based fluorescence lifetime imaging with synchronous external illumination: A new path for clinical translation. *J. Biophotonics* **13** (2020). 10.1002/jbio.201960119 31742905

[47] Lagarto JL (2020). Autofluorescence Lifetime Reports Cartilage Damage in Osteoarthritis. Sci. Rep.

[48] Bronzi D (2014). 100 000 frames/s 64 × 32 single-photon detector array for 2-D imaging and 3-D ranging. IEEE J. Sel. Top. Quantum Electron..

[49] Bronzi D (2016). Automotive Three-Dimensional Vision Through a Single-Photon Counting SPAD Camera. IEEE Trans. Intell. Transp. Syst..

[50] Scully AD (1996). Development of a laser-based fluorescence microscope with subnanosecond time resolution. J. Fluoresc..

[51] Gerritsen HC, Sanders R, Draaijer A, Ince C, Levine YK (1997). Fluorescence lifetime imaging of oxygen in living cells. J. Fluoresc..

[52] Fereidouni, F., Esposito, A., Blab, G. A. & Gerritsen, H. C. A modified phasor approach for analyzing time-gated fluorescence lifetime images. *J. Microsc*. (2011). 10.1111/j.1365-2818.2011.03533.x 21933184

[53] Manning HB (2013). Detection of cartilage matrix degradation by autofluorescence lifetime. Matrix Biol..

[54] Gorpas, D. *et al*. Autofluorescence lifetime augmented reality as a means for real-time robotic surgery guidance in human patients. 1–9, 10.1038/s41598-018-37237-8 (2019). 10.1038/s41598-018-37237-8 PMC6362025 30718542

[55] Alfonso-Garcia, A. *et al*. Real-time augmented reality for delineation of surgical margins during neurosurgery using autofluorescence lifetime contrast. *J. Biophotonics* 1–12, 10.1002/jbio.201900108 (2019). 10.1002/jbio.201900108 PMC7510838 31304655

[56] Cheng S (2014). Handheld multispectral fluorescence lifetime imaging system for *in vivo* applications. Biomed. Opt. Express.

[57] Felson DT (1988). Epidemology of hip and knee osteoarthritis. Epidemliologic Rev.

[58] Pearle AD, Warren RF (2005). & Rodeo, S. a. Basic science of articular cartilage and osteoarthritis. Clin. Sports Med..

[59] Murphy G, Nagase H (2008). Reappraising metalloproteinases in rheumatoid arthritis and osteoarthritis: destruction or repair?. Nat. Clin. Pract. Rheumatol..

[60] Manning HB (2008). A compact, multidimensional spectrofluorometer exploiting supercontinuum generation. J. Biophotonics.

[61] Zhou, X. *et al*. Detection of glycosaminoglycan loss in articular cartilage by fluorescence lifetime imaging cartilage by fluorescence lifetime imaging. **23** (2019). 10.1117/1.JBO.23.12.126002 PMC8357192 30578627

[62] Colyer RA, Lee C, Gratton E (2008). A Novel Fluorescence Lifetime Imaging System That Optimizes Photon Efficiency. Microsc. Res. Tech..

[63] Lagarto, J., Hares, J. D., Dunsby, C. & French, P. M. W. Development of Low-Cost Instrumentation for Single Point Autofluorescence Lifetime Measurements. *J. Fluoresc*., 10.1007/s10895-017-2101-7 (2017). 10.1007/s10895-017-2101-7 PMC5583312 28540652

[64] Scheeline A (2017). How to Design a Spectrometer. Appl. Spectrosc..

[65] Cicchi R (2014). Combined fluorescence-Raman spectroscopic setup for the diagnosis of melanocytic lesions. J. Biophotonics.

[66] Sherlock BE, Phipps JE, Bec J, Marcu L (2017). Simultaneous, label-free, multispectral fluorescence lifetime imaging and optical coherence tomography using a double-clad fiber. Opt. Lett..

