# Supplementary material for: Real-time multispectral fluorescence lifetime imaging using Single Photon Avalanche Diode arrays
Source: Sci Rep. 2020 May 15;10:8116. doi: 10.1038/s41598-020-65218-3 (PMC7229199; doi:10.1038/s41598-020-65218-3)
Supplement: Supplementary file 1 — Supplementary Information. [file 41598_2020_65218_MOESM1_ESM.pdf]

Real-time multispectral fluorescence lifetime imaging using Single Photon Avalanche Diode arrays

João L. Lagarto[1,2,*], Federica Villa[3], Simone Tisa[4], Franco Zappa[3], Vladislav Shcheslavskiy[5], Francesco S. Pavone[1,2,6] and Riccardo Cicchi[2,3]

[1] National Institute of Optics National Research Council (INO-CNR), Largo Enrico Fermi 6, 50125, Florence, Italy

[2] European Laboratory for Non-linear Spectroscopy (LENS), Via Nello Carrara 1, 50019, Sesto Fiorentino, Italy

[3] Dipartimento di Elettronica, Informazione e Bioingegneria (DEIB), Politecnico di Milano, 20133 Milan, Italy

[4] Micro Photon Device SRL, Via Waltraud Gebert Deeg 3g, I-39100, Bolzano, Italy

[5] Becker & Hickl GmbH, Nunsdorfer Ring 7-9, 12277 Berlin, Germany

[6] Department of Physics, University of Florence, Via G. Sansone 1, 50019, Sesto Fiorentino, Italy

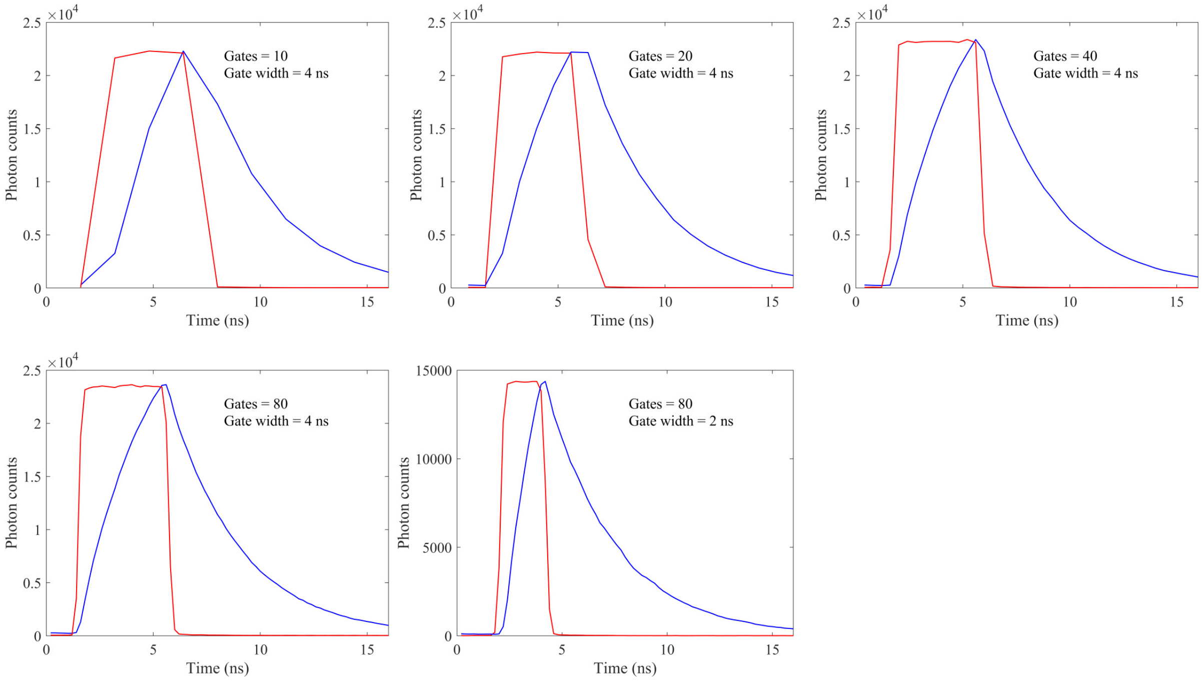

Figure S1. Instrument response function (in blue) and fluorescence intensity decay of a fluorescence standard (in red) for different time-gating configurations.

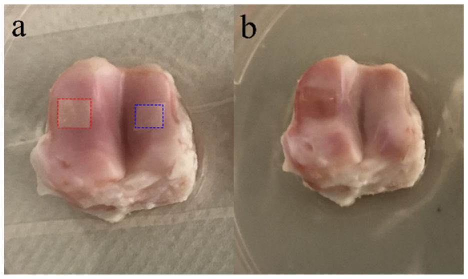

Figure S2. Cartilage samples (a) before and (b) after treatment. In (a), regions of interest in red and blue identify the locations where filter paper soaked in bacterial collagenase or PBS were applied, respectively.

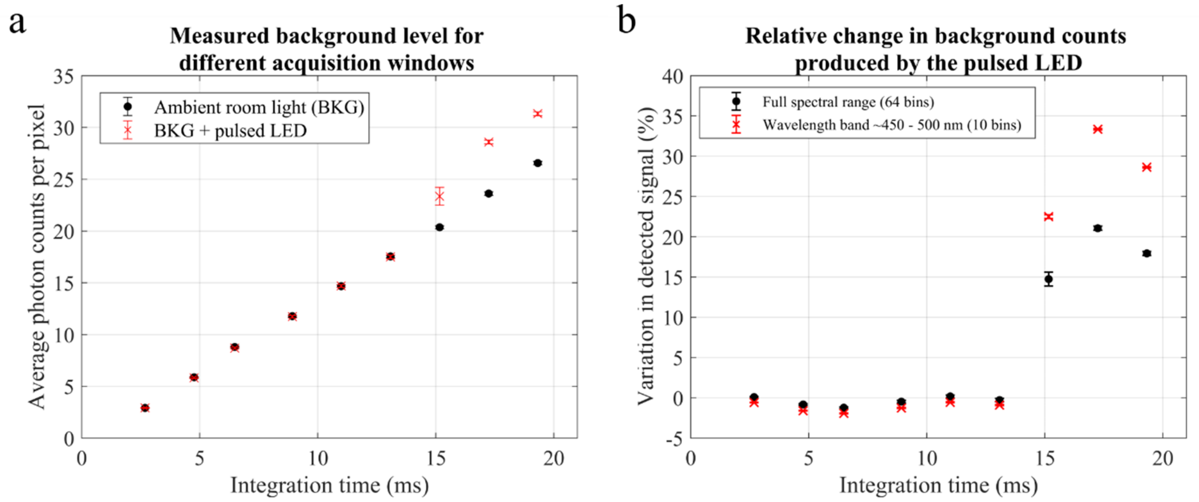

Figure S3. (a) Measured background levels at different integration windows when LED is off (black circles, measurements realized with ambient room light only) and when LED is pulsing at 50 Hz, interleaved with fluorescence lifetime acquisitions (red crosses); (b) Increase in measured background signal when the LED is pulsing relative to ambient light only. Output from pulsed LED was maintained constant throughout the measurements. A total of n = 200 measurements was realized for each integration window.

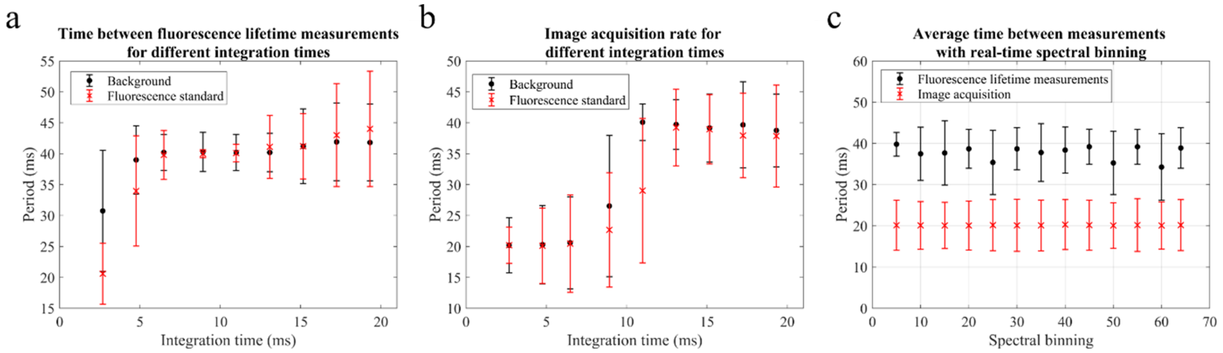

Figure S4. Fluorescence lifetime (a) and white light image (b) acquisition rates for different fluorescence integration times. While both fluorescence and white light image acquisition are triggered at 50 Hz, the measured lower frequency as a result of computational delays caused by fluorescence data and image processing. This is most noticeable at longer integration times, since the allotted interval for data analysis becomes increasingly shorter. (c) Effect of real-time spectral binning of data in fluorescence lifetime and color image acquisition rates. Integration time for fluorescence lifetime measurements was set to 11 ms. A total of n = 200 measurements was realized for each integration window. Frame exposure time in the color camera was set to 1.93 ms.

Fluorescence spectral and lifetime measurements of reference fluorophores

Stock solutions of flavin adenine nucleotide (FAD, F6625, Sigma-Aldrich, Saint Louis, MO, USA) and 1,4-bis(5-phenyloxazol-2-yl) benzene (POPOP, P3754, Sigma-Aldrich) were prepared by dissolving the corresponding powder in 50 mL of purified water and ethanol, respectively, to achieve concentrations of 50 µM. Two mixture solutions were obtained from stock solutions with the approximate proportions 1:2 and 2:1 ([FAD]:[POPOP]).

A key point of our implementation refers to the dynamic spectral lifetime resolution, which can be tuned according to the requirements of each application. In order to verify the impact of the spectral resolution in the fluorescence output, we measured the fluorescence lifetime and spectral characteristics of pure FAD and POPOP, and two mixture solutions (see Fig. S5). For pure solutions of POPOP (see Fig. S5(a, b), dark blue) and FAD (see Fig. S5 (a, b), dark green), we measured maximum fluorescence emission at ~425 nm and 530 nm, respectively, which are in good agreement with previous reports [1,2]. For the two mixture solutions, the fluorescence spectra show traces of both fluorophores in different proportions: when FAD is

predominant (light green), we measured maximum emission at ~530 nm and a shoulder at 410-430 nm consistent with POPOP emission peak; when POPOP is the predominant fluorophore (cyan), maximum emission occurs at 420 nm and is accompanied by a shoulder at 510-520 nm, which is indicative of FAD fluorescence.

Fluorescence lifetime data for each solution are presented in Fig. S5c, where each column depicts a different solution, with measurements of pure FAD and POPOP presented on the far left and right columns, respectively, and the two mixture solutions in the center. To demonstrate how spectral resolution impacts the fluorescence readout, data were spectrally binned in post-processing to adjust the spectral resolution of the measurements, from its maximum (Fig. S5c bottom row, 64 spectral channels, corresponding to 1 column of pixels per channel) to its minimum (Fig. S5c top row, 1 spectral channel, corresponding to 64 columns of pixels merged into a single channel). Each point in the phasor cloud represents a fluorescence decay along the spectrum, i.e. for a defined spectral band. Consequently, the greatest the spectral resolution, the larger the number of phasors in each plot. For pure FAD and POPOP (far left and far right columns, respectively), we obtained compact phasor clouds independently of the spectral resolution. This indicates that the fluorescence lifetimes of both FAD and POPOP are independent of the wavelength. In turn, for mixture solutions, the fluorescence specificity increases with spectral resolution. This is most evident when POPOP is the dominant fluorophores (see Fig. S5c, third column). Specifically, when data are merged into a single channel (Fig. S5c, panel 3), the corresponding fluorescence decay will be a weighted average of the fluorescence decays across the spectrum. Since POPOP is predominant over FAD (see Fig. S5b, cyan curve), the corresponding phasor will be closer to the pure POPOP phasor. If data are binned into four equal spectral channels (Fig. S5c, panel 7), the specificity of the measurement increases and two clusters of phasors are now evident in the phasor map, corresponding to the FAD and POPOP spectrally-resolved fluorescence signals. A third cluster is also visible along the line connecting the pure populations, representing fluorescence decays within the spectral region where POPOP and FAD have competing contributions, i.e. from ~450 nm to 500 nm. As the spectral resolution increases (see Fig. S5c, panels 11, 15 and 19), the number of phasor clusters along the line connecting the pure populations also increases and their distribution varies depending on the contribution of each fluorophore in each spectral band: fluorescence decays from short wavelength bands are shifted towards pure POPOP (to the right); in turn, fluorescence decays from long wavelength bands are closer to FAD (to the left). For maximum spectral resolution, i.e. 64 spectral channels, fluorescence decays in each channel have a slightly different contribution from each fluorophore, resulting in slightly different fluorescence lifetimes. Accordingly, the corresponding phasors occupy the entire region between FAD and POPOP pure populations, shifting from the pure POPOP towards FAD as the wavelength increases (Fig. S5c, panel 23).

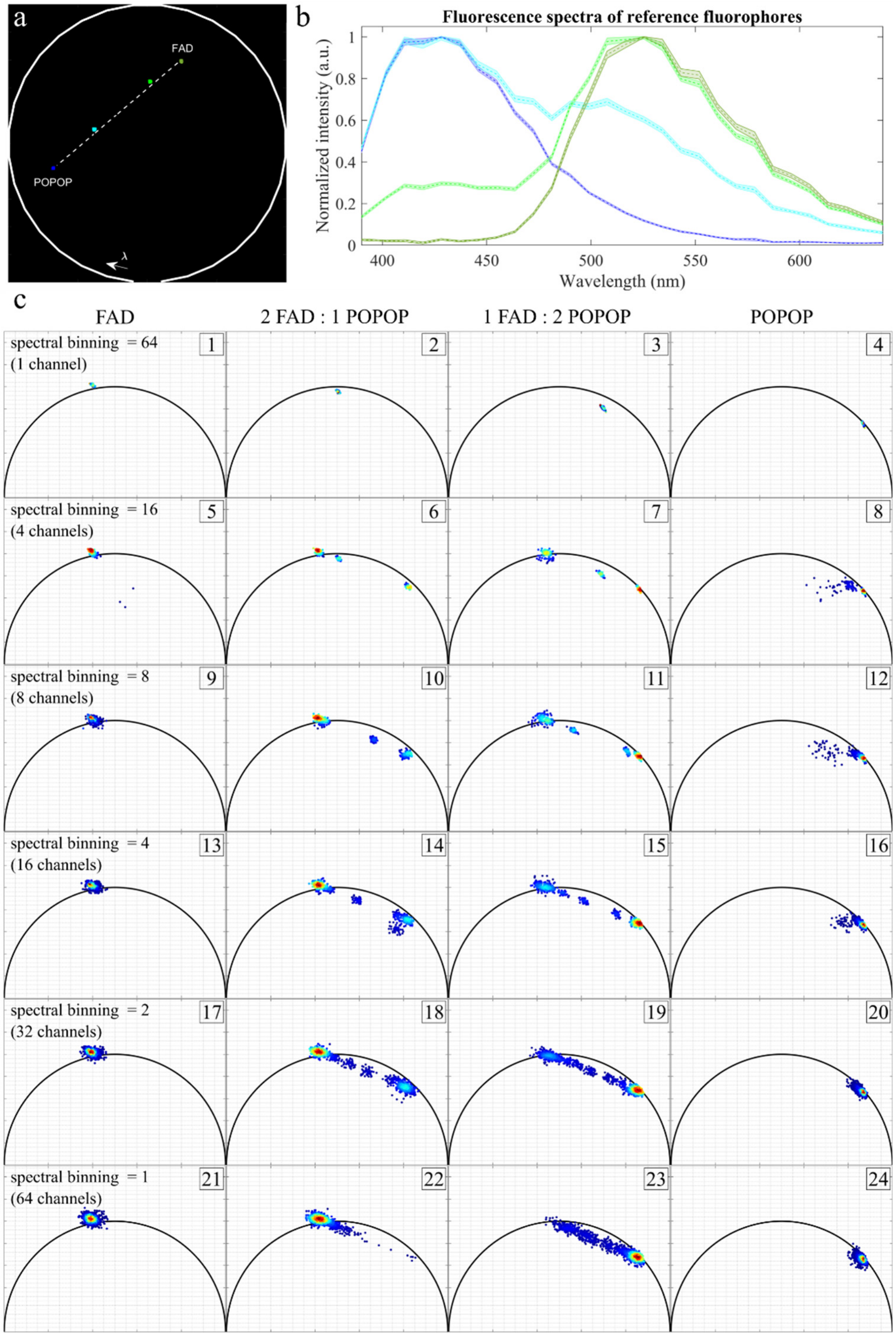

Figure S5. (a) Spectral phasor map and (b) fluorescence emission spectra of pure POPOP (in blue) and FAD (dark green), and mixture solutions at different concentrations (cyan and light green). (c) Fluorescence lifetime phasor maps for all solutions. Each column depicts a different solution with measurements of pure FAD and POPOP presented on the far left and right columns, respectively, and the two mixture solutions on the center. A total of 50 measurements were realized for each solution. Rows show post-processing results for different spectral binning. Each point in the phasor cloud represents a fluorescence decay along the spectrum. For example, top row illustrates the results of binning the entire spectrum in a single channel, resulting in a maximum of 50 phasor points (from n = 50 measurements) for each solution. Bottom row shows the results of no spectral binning i.e. 64 spectral channels, resulting in a maximum of 3200 phasor points per solution. Fluorescence decays with less than 100 photon counts at the peak were removed.

References

1. König, K.; Berns, M.W.; Tromberg, B.J. Time-resolved and steady-state fluorescence measurements of β-nicotinamide adenine dinucleotide-alcohol dehydrogenase complex during UVA exposure. *J. Photochem. Photobiol. B Biol.* **1997**, *37*, 91–95.

2. Islam, M.S.; Honma, M.; Nakabayashi, T.; Kinjo, M.; Ohta, N. pH Dependence of the Fluorescence Lifetime of FAD in Solution and in Cells. *Int. J. Mol. Sci.* **2013**, *14*, 1952–63.
